# Effect of low-level laser therapy on bisphosphonate-treated osteoblasts

**DOI:** 10.1186/s40902-016-0095-8

**Published:** 2016-11-25

**Authors:** Sang-Hun Shin, Ki-Hyun Kim, Na-Rae Choi, In-Ryoung Kim, Bong-Soo Park, Yong-Deok Kim, Uk-Kyu Kim, Cheol-Hun Kim

**Affiliations:** 1 Department of Oral and Maxillofacial Surgery, School of Dentistry, Pusan National University, Beomeo, Mulgeum, Yangsan, 626-770 Republic of Korea; 2 Department of Oral Anatomy and Cell Biology, School of Dentistry, Pusan National University, Beomeo, Mulgeum, Yangsan, 626-770 Republic of Korea; 3 Department of Oral and Maxillofacial Surgery, Dentistry, Dong-A Medical Center, 602-715 Pusan, Republic of Korea

**Keywords:** Bisphosphonate, Osteoblasts, Low-level laser therapy (LLLT), BRONJ

## Abstract

**Background:**

This study investigates the effect of alendronate-treated osteoblasts, as well as the effect of low-level laser therapy (LLLT) on the alendronate-treated osteoblasts. Bisphosphonate decreases the osteoblastic activity. Various treatment modalities are used to enhance the bisphosphonate-treated osteoblasts; however, there were no cell culture studies conducted using a low-level laser.

**Methods:**

Human fetal osteoblastic (hFOB 1.19) cells were treated with 50 μM alendronate. Then, they were irradiated with a 1.2 J/cm^2^ low-level Ga-Al-As laser (*λ* = 808 ± 3 nm, 80 mW, and 80 mA; spot size, 1 cm^2^; NDLux, Seoul, Korea). The cell survivability was measured with the MTT assay. The three cytokines of osteoblasts, receptor activator of nuclear factor κB ligand (RANKL), osteoprotegerin (OPG), and macrophage colony-stimulating factor (M-CSF) were analyzed.

**Results:**

In the cells treated with alendronate at concentrations of 50 μM and higher, cell survivability significantly decreased after 48 h (*p* < 0.05). After the applications of low-level laser on alendronate-treated cells, cell survivability significantly increased at 72 h (*p* < 0.05). The expressions of OPG, RANKL, and M-CSF have decreased via the alendronate. The RANKL and M-CSF expressions have increased, but the OPG was not significantly affected by the LLLT.

**Conclusions:**

The LLLT does not affect the OPG expression in the hFOB cell line, but it may increase the RANKL and M-CSF expressions, thereby resulting in positive effects on osteoclastogenesis and bone remodeling.

## Background

Bisphosphonates (BPs) are a group of medications that are known to be effective for inhibiting bone resorption. They have been used in clinical settings for over 30 years [1] in patients with multiple myeloma, metastatic skeletal disease, and hypercalcemia of malignancy. BPs are also effective in patients with Paget’s disease of bone and osteoporosis [2].

First described by Marx in 2003, bisphosphonate-related osteonecrosis of the jaw (BRONJ) is a side effect of BP use in dentistry [3]. Many authors have hypothesized that BRONJ is related to the oversuppression of bone turnover caused by BP usage [4]. Subramaian et al. [5] introduced a hypothesis that focuses on a defective remodeling process secondary to weakened synergism among the key cell types that interact during bone remodeling, including osteoblasts, osteoclasts, osteocytes, and bone lining cells.

At the cellular level, BPs have been known to act directly or indirectly on the osteoclasts. Decreased osteoclastogenesis can occur either directly on osteoclast precursors or indirectly by stimulating the osteoblasts to produce an inhibitor of osteoclast formation. The inhibition of the osteoblast-osteoclast pathway is an important component of the bisphosphonate activity [6].

The bone is continuously destroyed and reformed by a strictly regulated equilibrium between osteoblastic bone formation and osteoclastic resorption. During this process, osteoblasts stimulate bone formation and mediate osteoclast differentiation and function via cell-to-cell contact with osteoclast precursors. The roles of various substances related to the bone resorption process were recently clarified. These substances include the receptor activator of nuclear factor κB ligand (RANKL), osteoprotegerin (OPG), and macrophage colony-stimulating factor (M-CSF).

Researchers have focused on additional methods to support healing in the management of BRONJ, including medications and surgical procedures. An additional supporting method is low-level laser therapy (LLLT) [7]. Yaakobi et al. [8] reported the curative effects of LLLT in bone regeneration. In vitro laser biostimulation studies have also been performed with osteoblasts or osteoblast-like cells. Dörtbudak et al. [9] reported that LLLT has stimulatory effects on the bone matrix formation in osteoblast cell culture. However, the effects of LLLT on bisphosphonate-treated osteoblasts are not well known.

This study investigated the expressions of RANKL, OPG, and M-CSF in bisphosphonate-treated osteoblasts, as well as the effects of LLLT on bisphosphonate-treated osteoblasts, in order to provide an experimental basis for BRONJ treatment.

## Methods

### Cell culture and treatment with alendronate

A human fetal osteoblast (hFOB 1.19) cell line was purchased from the American Type Culture Collection (ATCC; Rockville, MD, USA). The hFOB 1.19 cells were cultured at 34 °C with 5% CO_2_ in an incubator. The culture medium was a 1:1 mixture of Dulbecco/Vogt modified Eagle’s minimal essential medium (DMEM) and F12 (Invitrogen, USA) supplemented with 10% fetal bovine serum (Invitrogen). Cells were cultured on culture dishes and/or in several types of wells for 24 h, after which the original medium was removed and the cells were washed with phosphate-buffered saline (PBS). Alendronate (Sigma, St. Louis, MO, USA) stock solution was added to the fresh medium in order to attain drug concentrations of 0, 25, 50, 100, 250, 500, and 1000 μM.

### Low-level laser irradiation

After the alendronate treatment, laser irradiation was performed with a gallium-arsenide-aluminum (Ga-Al-As) laser (*λ* = 808 ± 3 nm; 80 mW; 80 mA; spot size, 1 cm^2^; NDLux; Seoul, Korea) in the dark to eliminate the influence of other light sources. Laser energy was provided to the cells in continuous mode and vertical direction of each well. The laser handpiece was fixed, and the plate was moved to irradiate one well at a time (5 cm above the bottom of the culture plate). Laser was applied for a duration of 15 s for each well at 0, 24, and 48 h. After 72 h, the total irradiated energy was 3.6 J/cm^2^.

### Cell survivability assay

A total of 1 × 10^4^ cells were seeded in a 96-well plate, incubated for 24 h, and treated with alendronate at various concentrations and time points with and without low-level laser irradiation. Then, the cells were treated with 500 μg/ml of MTT stock solution and incubated at 34 °C in a 5% CO_2_ atmosphere for 4 h. The medium was aspirated, and the formazan crystals were dissolved in DMSO. Cell survivability was monitored on an ELISA reader (Tecan, Männedorf, Switzerland) at an excitation emission wavelength of 570 nm.

### Western blot analysis

The Western blot analysis was performed by using mouse sRANKL, OPG, M-CSF, and rabbit polyclonal anti-human GAPDH antibody (Santa Cruz Biotechnology, Santa Cruz, CA, USA).

The cells were washed twice with ice-cold PBS and centrifuged at 2000 rpm for 10 min. The total cell proteins were lysed with a RIPA buffer (300 mM NaCl, 50 mM Tris-HCl [pH 7.6], 0.5% TritonX-100, 2 mM PMSF, 2 μg/ml aprotinin, and 2 μg/ml leupeptin) and incubated at 4 °C for 1 h. The lysates were centrifuged at 14,000×*g* at 4 °C for 15 min, and sodium dodecyl sulfate (SDS) and sodium deoxycholic acid (0.2% final concentration) were added. The protein concentrations of the cell lysates were determined by means of a Bradford protein assay (Bio-Rad, Richmond, CA, USA), and the bovine serum albumin (BSA) was used as the protein standard. A 20 μg protein sample from each well was separated and loaded onto a 10% SDS-PAGE. The gels were transferred to a PVDF (Amersham GE Healthcare, Little Chalfont, UK), and they reacted to each antibody. Immunostaining with antibodies was performed by using a Super Signal West Femto enhanced chemiluminescent substrate and detected by Alpha Imager HP (Alpha Innotech, Santa Clara, CA, USA). Equivalent protein loading was confirmed by Ponceau S staining.

### RNA isolation and real-time PCR

The hFOB cells were subjected to RNA extraction by using spin columns (RNeasy; QIAGEN, Hilden, Germany), according to the manufacturer’s instructions. RNA (2 μg) was reverse transcribed by using the RevertAid First-Strand Synthesis System kit for real-time polymerase chain reaction (Thermo Fisher Scientific, Pittsburgh, PA, USA), according to the manufacturer’s protocol. The cDNA was amplified with the SYBR Green PCR master mix kit (Applied Biosystems, Warrington, UK), and the PCR amplification was performed by using the Chromo4 Real-Time PCR Detection System (Bio-Rad Laboratories, Inc.). Running conditions were as follows: incubation at 95 °C for 3 min, and 40 cycles of incubation at 95 °C for 15 s and 60 °C for 30 s. After the last cycle, the melting curve analysis was performed at 55–95 °C intervals by incremental temperature increases of 0.5 °C.

### Detection of RANKL, OPG, and M-CSF by ELISA

The RANKL, OPG, and M-CSF secretions were measured with an ELISA kit (Quantikine, R&D Systems, MN, USA), according to the manufacturer’s instructions. Cultured hFOB 1.19 cells were uniformly seeded into 6-well culture dishes at a concentration of 2 × 10^5^ cells/well. When the cells became adherent after 24 h, the medium was replaced with a medium containing 50 μM alendronate for 48 h. Laser irradiation was subsequently applied three times at 0, 24, and 48 h. The supernatants were collected and ELISA was used to determine the RANKL, OPG, and M-CSF concentrations in each sample at 72 h. The values were determined based on a standard curve set to 450 nm with a 540–570-nm wavelength correction and expressed as pg/ml. All of the samples were simultaneously assayed.

### Statistical analysis

A comparative analysis was run by one-way ANOVA with Tukey’s post hoc test for cell survivability, RT-PCR, and ELISA (SPSS version 17.0). The *P* values <0.05 were considered as statistically significant.

## Results

### Cell survivability following the treatment with alendronate and low-level laser therapy

**Fig. 1:**
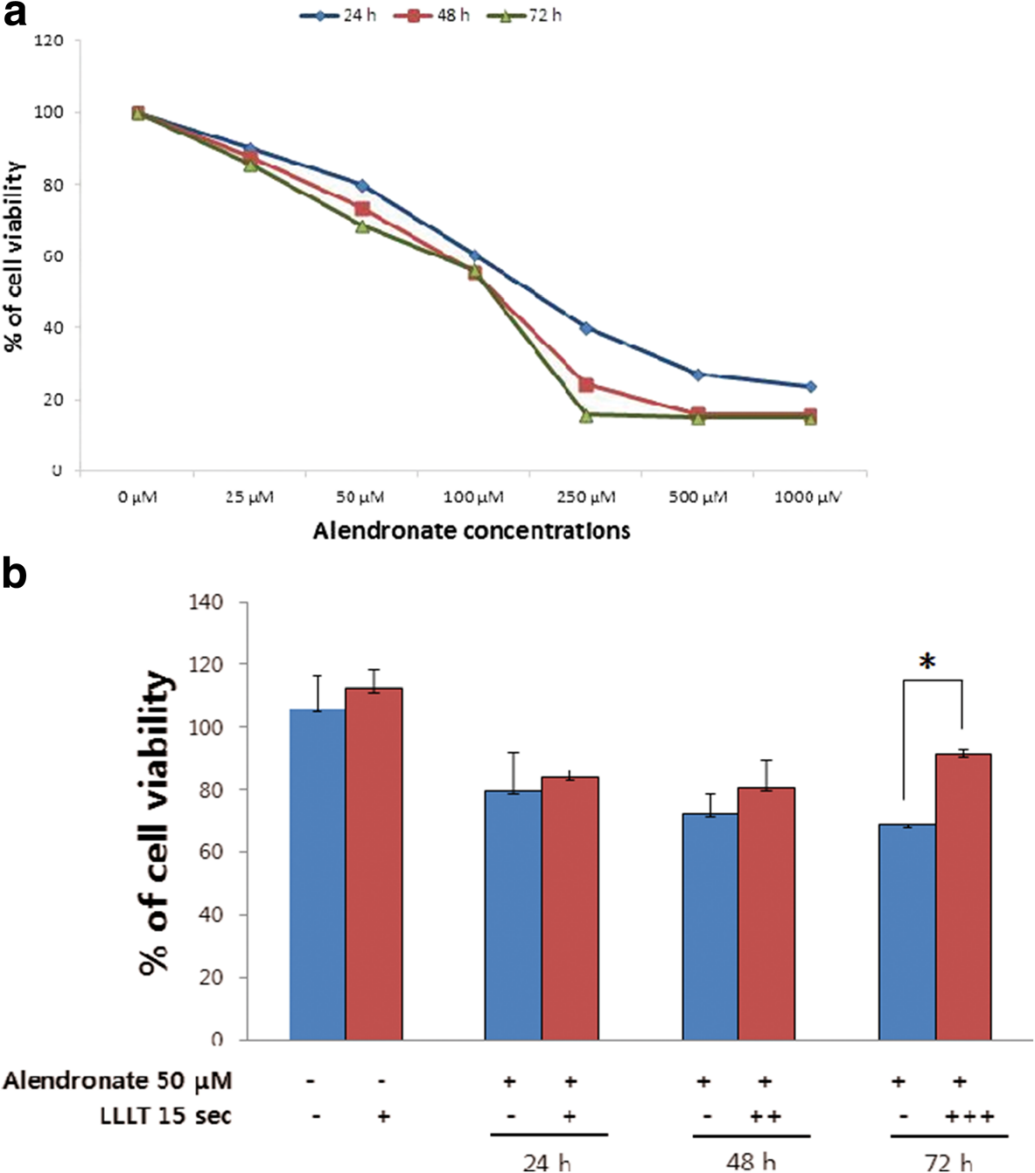
**a** Cell viability following treatment with different concentrations of alendronate and LLLT in hFOB 1.19 cells. **b** Cell viability following treatment with 50 μM alendronate and LLLT in hFOB 1.19 cells. (*Statistical significance (*p* < 0.05) compared with alendronate-treated group)

An hFOB cell survivability assay was performed at 24, 48, and 72 h with 0, 25, 50, 100, 250, 500, and 1000 μM alendronate concentrations (Fig. 1a). In the cells treated with alendronate at concentrations of 50 μM and higher, the cell survivability significantly decreased after 48 h (*p* < 0.05). In order to evaluate the effects of LLLT on alendronate-treated cells, the cell survivability was measured at 24, 48, and 72 h after the laser application (Fig. 1b).

The cell survivability in the alendronate-treated group continuously decreased. After three applications of low-level laser (LLL), the cell survivability significantly increased at 72 h (*p* < 0.05).

### Effects of alendronate and LLLT on OPG expression

**Fig. 2:**
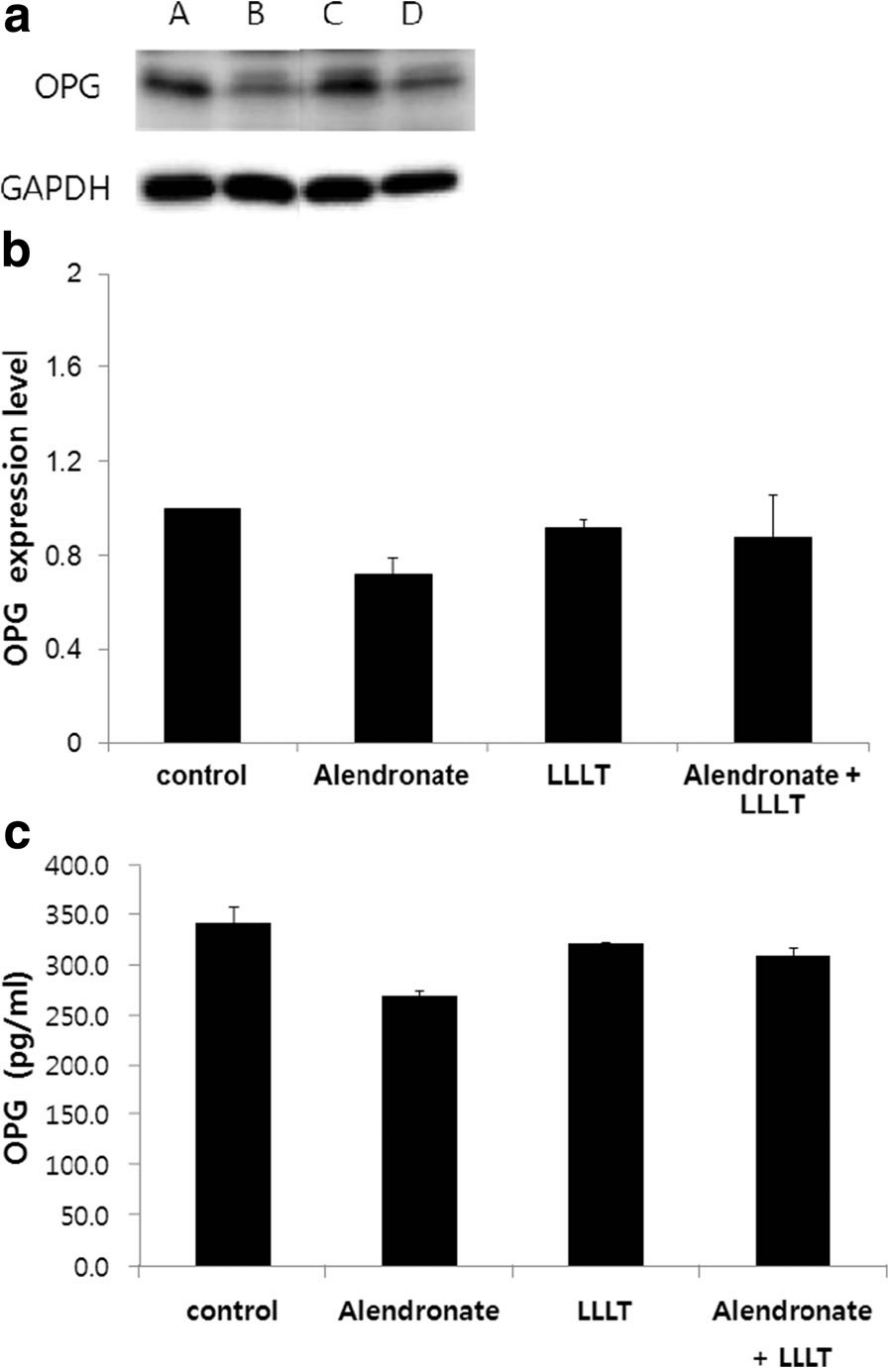
OPG expression in cells treated with alendronate and LLLT. **a** Western blot. **b** RT-PCR. **c** ELISA

Western blot, RT-PCR, and ELISA were used in order to determine the OPG expression. The OPG expression in the alendronate-treated cells decreased more than that of the control group; however, there was no statistical significance (Fig. 2b). In the alendronate cells treated with the laser, the OPG expression increased more than that of the alendronate-treated group; however, the difference was not statistically significant (Fig. 2b, c).

### Effects of alendronate and LLLT on RANKL expression

**Fig. 3:**
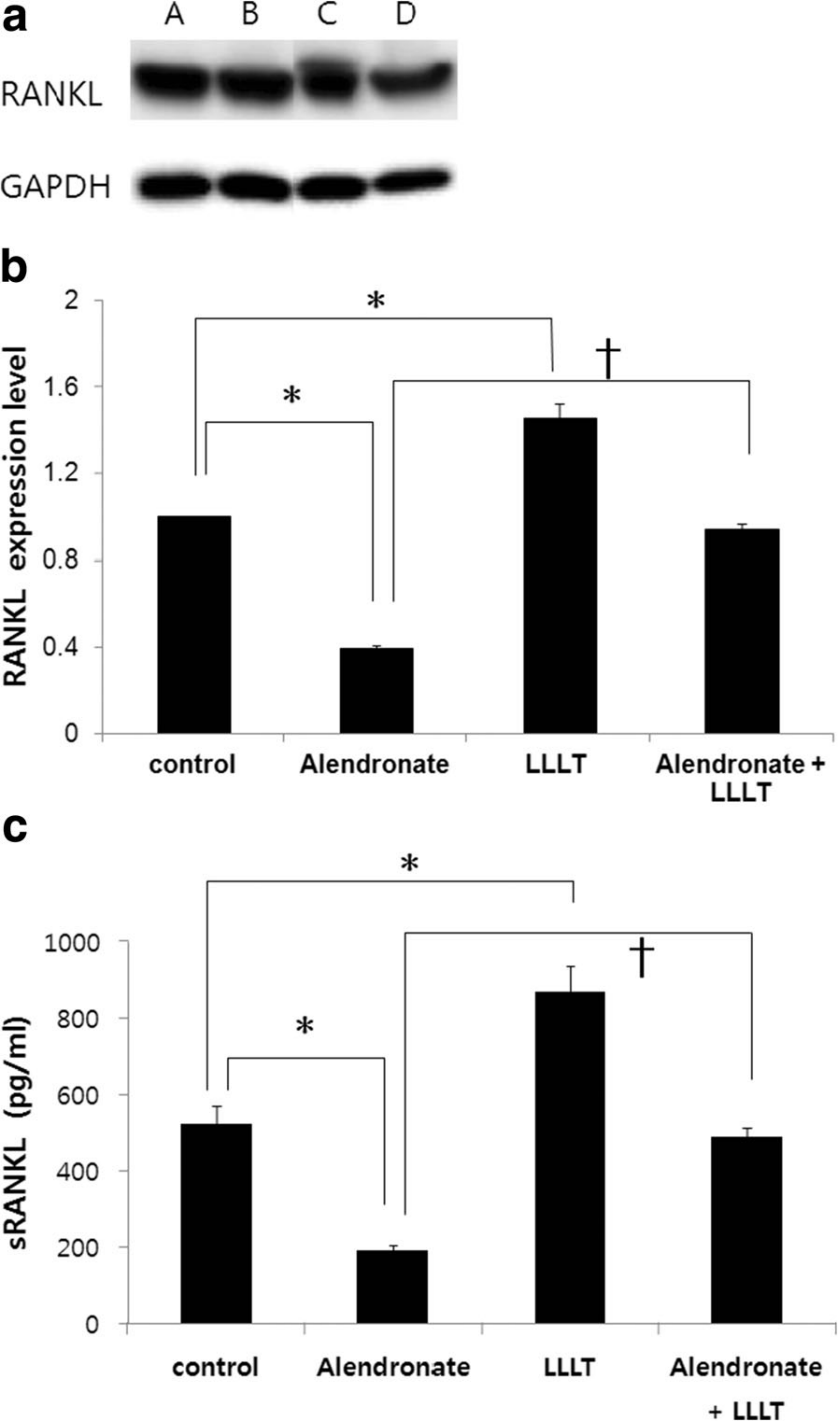
RANKL expression in cells treated with alendronate and LLLT. **a** Western blot. **b** RT-PCR. **c** ELISA

Western blot, RT-PCR, and ELISA were used in order to determine the RANKL expression, which induces osteoclastogenesis. In the alendronate-treated cells, the RANKL expression decreased more than that of the control group (Fig. 3b). In the alendronate cells treated with the laser, the RANKL expression increased more than that of the alendronate-treated group (Fig. 3b, c).

### Effects of alendronate and LLLT on M-CSF expression

**Fig. 4:**
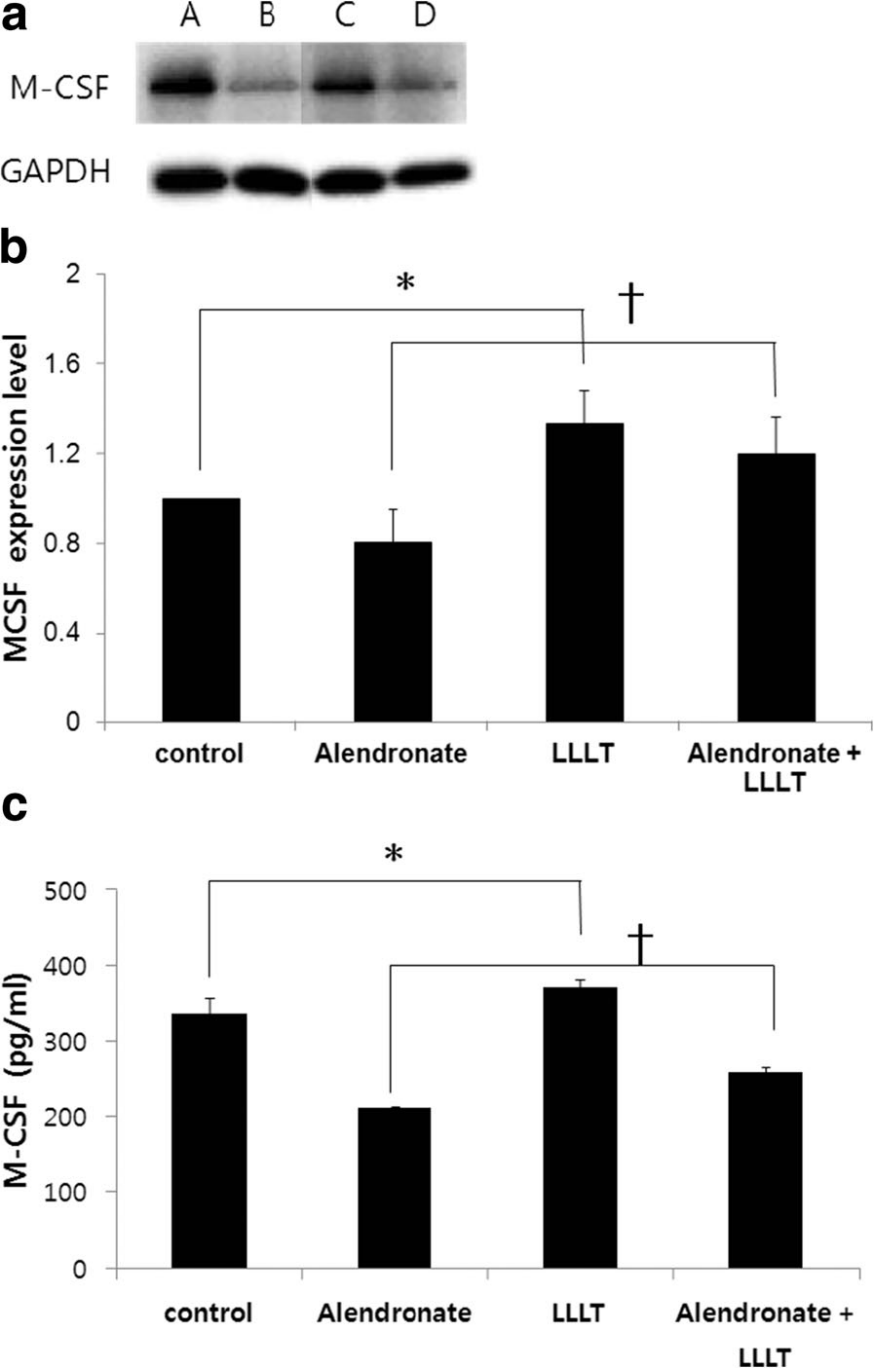
M-CSF expression in cells treated with alendronate and LLLT. **a** Western blot. **b** RT-PCR. **c** ELISA

Western blot, RT-PCR, and ELISA were performed in order to investigate the M-CSF expression, which is another factor related to osteoclastogenesis. In the cells treated with alendronate, the M-CSF expression decreased more than that of the control group; however, there was no statistical significance (Fig. 4b). In the alendronate cells treated with the laser, the M-CSF expression increased more than that of the alendronate-treated group (Fig. 4b, c).

## Discussion

BPs are the most widely used anti-resorptive drugs for metabolic bone diseases [10]. BPs are pyrophosphate-like structures with two variable regions (R1 and R2) on the carbon atom of the BP molecule attached to the basic P-C-P structure. This allows for variations in the molecular structure and a range of corresponding potencies. BPs are classified according to the chemical group added to the base pyrophosphoric nucleus at the R2 side chain. Alkyl derivatives are the first generation of drugs (e.g., etidronate). The second generation includes amino-bisphosphonates with a terminal amino group (e.g., alendronate and pamidronate), while the third generation is characterized by a cyclic side chain (e.g., zoledronate). Depending on whether or not nitrogen is attached to the R2 side chain, BPs are classified as nitrogen-containing BPs (NBPs) or non-nitrogen-containing BPs (NNBPs). NBPs (e.g., pamidronate, alendronate, risedronate, ibandronate, and zoledronate) inhibit farnesyl pyrophosphate synthase (FPPS), which is an enzyme in the mevalonate pathway, and block the prenylation of small GTPase-signaling proteins. This results in the accumulation of active unprenylated GTPases in the cytoplasm of the osteoclast, which causes an inappropriate activation of the downstream signaling pathways, thereby leading to the disruption of normal osteoclast function and survival. As a result, NBPs suppress bone resorption through a direct effect on the osteoclasts and their precursors [11, 12].

In the present study, we examined the effects of alendronate on osteoblasts. Alendronate is the most widely prescribed oral BP, which is more likely to cause BRONJ. However, the precise mechanism of alendronate in BRONJ remains elusive [13].

The effects of BPs on osteoclasts are well understood, and their toxicity effects on osteoclast are thought to influence the onset of BRONJ. Besides the inhibition of osteoclasts, many complicated events may be related to BRONJ development, and interactions among the bone cells must be considered as a whole [14]. Although the majority of in vitro BP studies have focused on the activities of the osteoclast lineage cells, recent studies have suggested that the presence of osteoblastic family cells is required in order for the anti-resorptive effects of BPs to occur. This effect may depend upon soluble factors that are secreted by the osteoblasts, which inhibit the formation and activity of the osteoclasts [15]. RANKL, OPG, and M-CSF are essential factors that are produced by the osteoblast/stromal cells for osteoclast-osteoblast interactions. RANKL is an important factor in protecting bone resorption, extending the life of osteoclasts, and promoting differentiation. OPG is known as a decoy receptor protein that prohibits osteoclast activation by protecting the function of the receptor activator nuclear factor-kB (RANK), which is involved in osteoclast differentiation by combining with RANKL [16]. The role of OPG is largely associated with an initiation phase, in which OPG counteracts the osteoclastogenic activity of RANKL. During bone formation, osteoclast differentiation is suppressed through the OPG that is produced by the osteoblast [17]. Osteoblasts produce M-CSF, which is required for cell survival in the macrophage-osteoclast lineage, and the control of cell migration and reorganization [18]. However, studies on the effects of BPs on osteoblasts are the subject of debate. In addition, the effects of BPs on osteoblastic activities have been sparsely investigated in terms of BRONJ development [19–21]. Knowledge regarding the effects of alendronate on the hFOB cells, particularly in the OPG/RANKL system, is lacking.

In order to address this data gap, we investigated the expressions of RANKL, OPG, and M-CSF in BP-treated hFOB cells. In the cells treated with alendronate at concentrations of 50 μM and higher, the cell survivability significantly decreased after 48 h, and there was a strongly negative dose-dependent influence on the viability of the osteoblasts and induced toxic effects in the hFOB cells. Enjuanes et al. [12] reported that high concentrations of alendronate inhibited osteoblast proliferation in the primary hFOB cells and indicated that the drug did not significantly inhibit proliferative effects, as compared to controls at lower concentrations (≤10–5 M). Naidu et al. [22] reported that high concentrations of alendronate and zoledronate were cytotoxic and decreased the cell survivability at 72 h, and cytotoxicity leading to cell death was likely to result in osteonecrosis. Our results are similar to these studies and suggest that alendronate at higher concentrations, more than 50 μM will affect hFOB cell proliferation and viability significantly after 48 h. We observed that 50 μM alendronate appeared to suppress M-CSF and RANKL expressions, and decreased the OPG expression, as compared to the control group. The RANKL expression appeared to be more suppressed than the OPG expression. RANKL expression level was more decreased than OPG or M-CSF in RT-PCR and ELISA result. Appeared by Western blot analysis, OPG, RNAKL, and M-CSF proteins were decreased in alendronate-treated cells.

Therefore, we proposed that the BPs interfere with osteoclastogenesis through regulating mediators (e.g., M-CSF, RANKL, and OPG) by inhibition of their expressions. However, Lin et al. [23] found no significant influence on osteoblast RANKL and OPG gene expressions during a 48-h experimental period when investigating alendronate and pamidronate. In contrast, Mackie et al. [24] reported that the RANKL gene expression was inhibited, while the OPG gene expression was not altered by stimulation with pamidronate in an osteosarcoma cell line for 6 days. Although the reasons for these differences have not been completely explained, the distinct effects of various BPs (e.g., pamidronate, zoledronate, and alendronate) and the use of different cell lines (e.g., human vs. rat and primary vs. cancer) could play a role [25]. Additional in-depth studies will be required in order to understand the reasons for these differences.

In medicine and dentistry, diode lasers have been used predominantly in applications that are broadly termed as LLLT or biostimulation [26], and many studies have evaluated the therapeutic effects of LLLT on a broad range of disorders. LLLT applications, which have been promoted by some authors and manufacturers of the LLLT devices, included the acceleration of wound healing, enhancement of the remodeling and repair of bones, restoration of normal neural function following injury, pain attenuation, and modulation of the immune system [27]. Recently, research on the use of LLLs in dentistry has proceeded gradually, and the range of clinical applications has been extended. The term LLL includes soft lasers, mid-lasers, low-energy lasers, and cold lasers. A new international definition considers LLLT to be laser therapies that do not increase tissue temperature over 36.5 °C or normal body temperature. The wavelength of such lasers is reported to be 500–1200 nm. Recent literatures regarding pre-osteoblast stimulation with red laser were reported [28–31]. We used a Ga–As–Al laser with a wavelength of 808 nm, which is within the prescribed range. It is still unclear as to which of the parameters has the greatest effect on therapeutic efficacy, even though there are information about total energy dose, energy density, and laser spectrum. In this study, the greatest biostimulatory effect was observed when a dose of 3.6 J/cm^2^ was used. This may be due to the use of different methodologies, such as different types of cells, experimental timings, and radiation distance.

Many previous studies have demonstrated that LLLT is optimal in tissues under a specific stress, such as hypoxia [32–34], diabetes [35–38], and nutritional deficit [39]. Other previous studies have shown that LLLT may enhance the osteogenic potential of osteoblasts, and may promote metabolic bone activity and bone remodeling [40, 41]. LLLT has recently been used as a supportive technique in BRONJ treatment. Clinical cases that describe the application of LLLT to treat BRONJ have been reported, based on in vivo and in vitro experimental studies that demonstrated a biostimulative effect [7, 42, 43].

Vescovi et al. [44] reported successful results by using a combined treatment in BRONJ patients with medication plus Er:YAG laser surgery and LLLT. However, there have been no cell culture studies designed to explain the positive results obtained with the LLLT biostimulation in combination with surgery in clinical BRONJ cases. The present study investigated the effects of LLLT in hFOB cells treated with alendronate. The cell survivability significantly increased at 72 h relative to the alendronate-treated cells after LLT application. Moreover, we observed a significant increase in RANKL and M-CSF expressions, as compared to the cells treated only with alendronate. The results showed that alendronate at a concentration of 50 μM appeared to inhibit hFOB cell survivability, and suppress the M-CSF and RANKL expressions, and decreased the OPG expression, as compared to the control group. Moreover, the LLLT increased the RANKL and M-CSF expressions relative to the alendronate-treated cells.

In this study, the greatest biostimulatory effect was observed when a dose of 3.6 J/cm2 was used. It may be due to the use of different methodologies, such as different types of cells, experimental timings, and radiation distance. Recently, the choice for an appropriate laser source and standardization of radiation parameters will require further research in order to obtain an optimal result for a low-level laser study.

## Conclusions

The present study was conducted in order to determine if LLLT influences cell survivability and cellular functions in hFOB cells following alendronate exposure. The results showed that alendronate at concentrations of 50 μM and higher inhibited hFOB cell survivability. Alendronate at a concentration of 50 μM appeared to suppress the M-CSF and RANKL expressions, and decreased the OPG expression, as compared to the control group. Moreover, LLLT increased the RANKL and M-CSF expressions relative to the alendronate-treated cells, thereby resulting in positive effects on osteoclastogenesis. These results demonstrated the possibility of LLLT in partially overcoming the inhibitory effects of BP and provide an experimental basis to explain the clinical effects of LLLT as a potential treatment modality for BRONJ.
